# Nutritional Content of Ready-to-Eat Breakfast Cereals Marketed to Children

**DOI:** 10.1001/jamanetworkopen.2025.11699

**Published:** 2025-05-21

**Authors:** Shuoli Zhao, Qingxiao Li, Yuan Chai, Yuqing Zheng

**Affiliations:** 1 Department of Agricultural Economics, University of Kentucky, Lexington; 2 Department of Agricultural Economics and Agribusiness, Louisiana State University and LSU AgCenter, Baton Rouge; 3 GEMS Informatics Center, University of Minnesota, St Paul; 4 Department of Applied Economics, University of Minnesota, St Paul

## Abstract

This cross-sectional study examines trends in the nutritional composition of children’s ready-to-eat cereals introduced in the US market from 2010 to 2023.

## Introduction

Ready-to-eat (RTE) cereals are the predominant breakfast choice among US children.^1^ Although RTE cereals contribute to children’s nutrient intake,^2^ recent research and increasing public awareness highlight concerns about RTE cereals exceeding nutrition recommendations.^3^ Given their widespread consumption and potential impact on childhood nutrition, understanding trends in cereal composition is crucial for public health. This study examined trends in the nutritional composition of children’s RTE cereals introduced in the US market from 2010 to 2023.

## Methods

This cross-sectional study used data from the Mintel Global New Products Database, a comprehensive database that tracks new product launches for food and beverages. This database provides detailed information on product attributes, including nutritional content, ingredients, packaging, and target audience. We included all new children’s cereal products launched in the US market between January 1, 2010, and December 31, 2023, and children’s cereals were defined as RTE breakfast cereal products explicitly marketed to children aged 5 to 12 years (eg, through packaging or branding). The University of Kentucky institutional review board determined the study did not qualify as human participant research.

Primary outcome variables were total fat, sodium, total carbohydrates, sugar, protein, and dietary fiber per serving. Trends in nutrient content over time were assessed by calculating the mean value and 95% CI for each nutrient adjusted by serving size over the study period. Trends in these nutrient content over time were calculated with 2-sided *P* < .05 considered significant. This study followed the STROBE reporting guideline. All analyses were performed from October 2024 to November 2024, using Stata, version 15.6.

## Results

**Figure 1.**
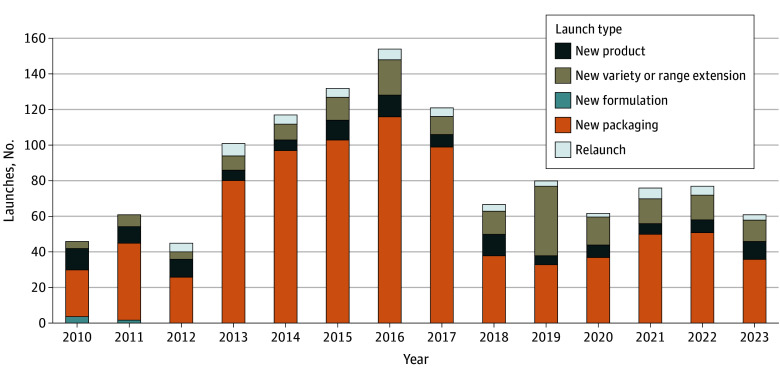
Number of Children’s Cereal Launches by Launching Type, 2010-2023 The bars in the figure show the yearly trend for new children’s cereal product launches. Each bar breaks down the relative frequency of launching types, including new formulations, new packaging, entirely new products, new variety or range extensions, and relaunches. Source: Mintel Global New Products Database, children’s cereal products launched January 2010 to December 2023.

**Figure 2.**
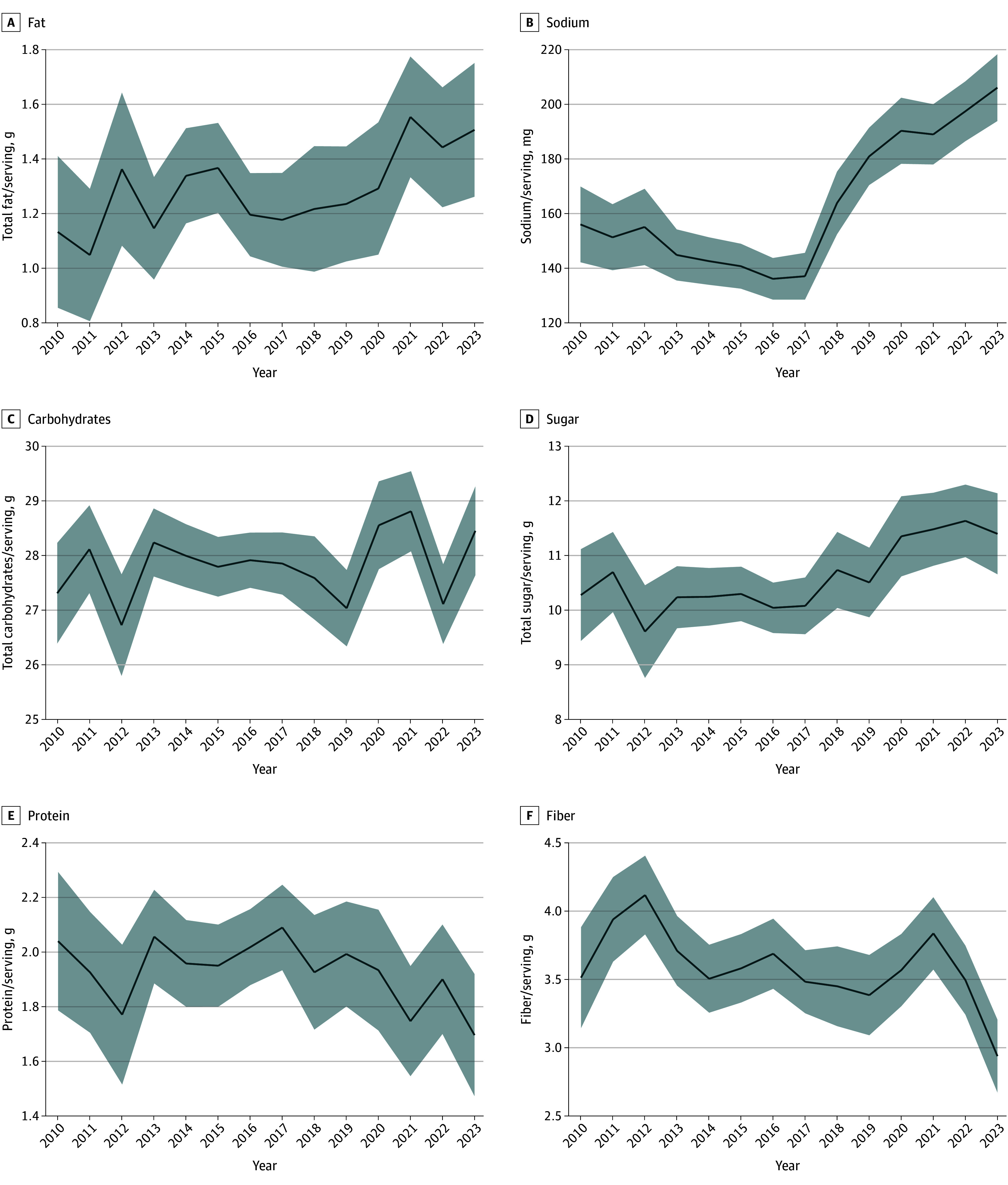
Trends in Nutritional Content Per Serving in New Children’s Cereals Launched in the US Market, 2010-2023 Solid blue lines indicate estimated mean nutrient values over time, and the shaded areas indicate 95% CIs. Nutrient values are reported per serving after adjusting for serving size. The y-axes differ to accommodate varying measurement scales for each nutrient. Source: Mintel Global New Products Database, children’s cereal products launched January 2010 to December 2023.

From 2010 to 2023, 1200 children’s RTE cereals in the US market were newly launched. Figure 1 illustrates the yearly trend in new children’s cereal product launches, showing that launching activity peaked around 2016. New packaging was the most common type of launch, and new formulation was the least common. Total fat per serving showed a 33.6% increase from 1.13 g (95% CI, 0.86-1.41 g) in 2010 to 1.51 g (95% CI, 1.26-1.75 g) in 2023 (Figure 2). Sodium content exhibited a 32.1% increase during the study, increasing from 156.0 mg (95% CI, 142.1-169.8 mg) to 206.1 mg (95% CI, 193.9-218.3 mg). Total carbohydrates showed a relatively stable trend from 27.32 g (95% CI, 26.40-28.23 g) in 2010 to 28.45 g (95% CI, 27.64-29.26 g) in 2023. Sugar content increased by 10.9% from 10.28 g (95% CI, 9.44-11.12 g) in 2010 to 11.40 g (95% CI, 10.66-12.14 g) in 2023. Protein content, whereas it fluctuated from 2010 to 2020, with a mean of 1.97 g (95% CI, 1.78-2.16 g), decreased significantly to 1.69 g (95% CI, 1.47-1.92 g) in 2023. Dietary fiber showed a steady trend before 2021, followed by a decrease from 3.82 g (95% CI, 3.57-4.09 g) in 2021 to 2.94 g (95% CI, 2.67-3.20 g) in 2023.

Sodium and fat content showed the most pronounced increases during the study, whereas the mean carbohydrate content was 26.44 g (95% CI, 25.35-27.52 g) from 2010 to 2019 and 32.64 g (95% CI, 29.80-35.47 g) from 2020 to 2023 (*P* < .001 for trend). Sugar content showed a more modest increase from 2017 to 2022 before a slight decrease. Comparatively, protein and fiber, as important nutritional content for children, have decreased over time (Figure 2).

## Discussion

Analysis of newly launched children’s RTE cereals from 2010 to 2023 revealed concerning nutritional shifts: notable increases in fat, sodium, and sugar alongside decreases in protein and fiber. Children’s cereals contain high levels of added sugar, with a single serving exceeding 45% of the American Heart Association’s daily recommended limit for children.^4^ These trends suggest a potential prioritization of taste over nutritional quality in product development, contributing to childhood obesity and long-term cardiovascular health risks.^5,6^ This study is limited in that it covered newly released cereals and thus does not represent the entire cereal market, nor does it allow for an assessment of the impact on children’s overall nutrient intake.

## Notes

Supplement.
Data Sharing Statement

## References

[1] Terry AL, Wambogo E, Ansai N, Ahluwalia N. Breakfast intake among children and adolescents: United States, 2015–2018. NCHS Data Brief. 2020;(386):1-8. 33054919

[2] Albertson AM, Anderson GH, Crockett SJ, Goebel MT. Ready-to-eat cereal consumption: its relationship with BMI and nutrient intake of children aged 4 to 12 years. J Am Diet Assoc. 2003;103(12):1613-1619. doi:10.1016/j.jada.2003.09.020 14647087

[3] Schwartz MB, Vartanian LR, Wharton CM, Brownell KD. Examining the nutritional quality of breakfast cereals marketed to children. J Am Diet Assoc. 2008;108(4):702-705. doi:10.1016/j.jada.2008.01.003 18375229

[4] Vos MB, Kaar JL, Welsh JA, ; American Heart Association Nutrition Committee of the Council on Lifestyle and Cardiometabolic Health; Council on Clinical Cardiology; Council on Cardiovascular Disease in the Young; Council on Cardiovascular and Stroke Nursing; Council on Epidemiology and Prevention; Council on Functional Genomics and Translational Biology; and Council on Hypertension. Added sugars and cardiovascular disease risk in children: a scientific statement from the American Heart Association. Circulation. 2017;135(19):e1017-e1034. doi:10.1161/CIR.0000000000000439 27550974 PMC5365373

[5] Yang Q, Zhang Z, Kuklina EV, . Sodium intake and blood pressure among US children and adolescents. Pediatrics. 2012;130(4):611-619. doi:10.1542/peds.2011-3870 22987869 PMC9011362

[6] Macé K, Shahkhalili Y, Aprikian O, Stan S. Dietary fat and fat types as early determinants of childhood obesity: a reappraisal. Int J Obes (Lond). 2006;30(4)(suppl 4):S50-S57. doi:10.1038/sj.ijo.0803519 17133236

