# Supplementary material for: Nutritional Content of Ready-to-Eat Breakfast Cereals Marketed to Children
Source: JAMA Netw Open. 2025 May 21;8(5):e2511699. doi: 10.1001/jamanetworkopen.2025.11699 (PMC12096261; doi:10.1001/jamanetworkopen.2025.11699)
Supplement: Supplement. — Data Sharing Statement [file jamanetwopen-e2511699-s001.pdf]

Data Sharing Statement

Zhao. Nutritional Content of Ready-to-Eat Breakfast Cereals Marketed to Children. *JAMA Netw Open*. Published May 21, 2025. doi:10.1001/jamanetworkopen.2025.11699

Data
Data available: No

Additional Information
Explanation for why data not available: The data used in this analysis is from Mintel Global New Product Database. Due to the proprietary nature of this data and the licensing agreement under which it was obtained, we are unable to share the data at the same detailed level. Aggregated data sufficient to reproduce the results are available upon request.
